# Electrical and mechanical properties of conductive elastic bands as wearable sensors

**DOI:** 10.1038/s41598-026-54874-6

**Published:** 2026-06-04

**Authors:** Eman Mustafa, Mohamed Naeem, Alaa Arafa Badr, Z. M. Abdel-Magied, Aliaa A. Mohamed

**Affiliations:** 1 Textile Department, Faculty of Technology and Education, Suez University, Suez, Egypt; 2 Faculty Dean, Faculty of Industrial and Energy Technology, Borg El Arab Technological University (BATU), Alexandria, Egypt; 3 Textile Engineering Department, Faculty of Engineering, Alexandria University, Alexandria, Egypt; 4 Clothing and Knitting Industrial Research Department, Textile Research and Technology Institute, National Research Centre, Giza, Egypt; 5 Textile and Clothing Department, Faculty of Women, Ain Shams University, Cairo, Egypt

**Keywords:** Crochet elastic bands, Wearable textile sensors, Dynamic breathing simulation, Abrasion resistance, Electrical stability, Engineering, Materials science

## Abstract

Crochet-based elastic textiles represent a promising option for wearable sensors due to their good stretchability and flexible structure. However, achieving cyclic electrical stability under realistic physiological motion and mechanical wear remains a critical challenge. This study systematically investigates the mechanical, aesthetic, and electrical performance of crochet elastic bands integrated with silver-coated polyamide (Ag/nylon) conductive yarns for wearable sensing applications. Dynamic resistance measurements were conducted using a custom-designed breathing simulator that replicates human respiratory motion, enabling real-time evaluation under cyclic deformation conditions. Conductive yarns were incorporated at different structural positions within the elastic bands to examine the influence of yarn configuration on sensor durability and signal stability. To simulate the cyclic use, samples were subjected to controlled abrasion cycles of 0, 5000, and 10,000 cycles. The results demonstrate that increasing abrasion levels lead to a gradual reduction in tensile strength and elongation due to progressive changes in surface morphology and stitch regularity, resulting in modified electrical pathways with the formation of pilling. Electrical performance strongly depended on conductive yarn placement. While several configurations exhibited significant signal degradation or delayed failure under abrasion, a specific configuration featuring balanced warp-weft integration of conductive yarns showed exceptional electrical stability, maintaining nearly constant resistance response across all abrasion levels. Statistical analysis confirmed the significant effects of both yarn configuration and abrasion cycles (*p* < 0.05). These findings highlight the critical role of conductive yarn positioning in enhancing the durability and sensing reliability of crochet-based wearable sensors. The proposed design strategy provides valuable guidance for the development of robust wearable sensors capable of maintaining stable electrical performance under simulated physiological motion and cyclic mechanical abrasion.

## Introduction

Wearable sensing technologies have gained increasing research interest due to their wide range of applications in healthcare monitoring, rehabilitation, and smart textile systems^1–4^. Among the various sensing approaches, textile-based strain sensors have emerged as a promising solution owing to their flexibility, lightweight nature, air permeability, and ability to adapt to the human body during daily activities^5,6^. These advantages make textile sensors particularly suitable for continuous physiological signal monitoring, such as respiration, motion, and posture analysis^7–10^.

Elastic textile structures are especially attractive for strain-sensing applications because they can undergo large deformations while maintaining structural integrity^11,12^. Crochet-based elastic bands offer additional benefits due to their looped construction, which allows controlled stretchability and recovery under cyclic loading conditions. When conductive yarns are incorporated into such elastic structures, mechanical deformation can be translated into detectable electrical resistance changes, enabling strain-sensing functionality^13,14^. However, ensuring stable electrical signals alongside sufficient mechanical durability remains a significant challenge for wearable textile sensors.

Previous studies have shown that the sensing performance of textile strain sensors is highly dependent on several parameters, including the type of conductive material, yarn linear density, fabric geometry, and integration method^15–19^. Silver-coated polyamide (Ag/nylon) yarns are widely used in wearable electronics due to their high conductivity, flexibility, and relatively good resistance to corrosion^20–22^. Despite these advantages, repeated mechanical deformation and surface abrasion can cause micro-level damage to the conductive coating, leading to increased electrical resistance and reduced sensing reliability over time^23–26^.

Abrasion and cyclic loading are unavoidable during real-life use of wearable sensors, especially in applications involving repetitive body movements such as breathing. Several researchers have reported that prolonged mechanical stress may result in coating delamination, yarn breakage, and loss of elastic recovery, ultimately affecting both mechanical strength and electrical stability^27^. Nevertheless, many existing studies focus primarily on material selection, while the influence of conductive yarn placement within elastic textile structures has received comparatively limited attention.

The positional arrangement of conductive yarns plays a crucial role in determining sensor behaviour, as it directly affects stress distribution, frictional contact, and inter-yarn electrical pathways during deformation^28^. Although knitted and woven textile strain sensors have been extensively studied, research on crochet-based elastic sensors remains scarce, particularly regarding their electrical performance under abrasion conditions. Moreover, most reported testing methods rely on static or simplified loading, which does not accurately represent realistic physiological motions such as human respiration^29^.

To overcome these limitations, the present study investigates crochet elastic bands incorporating Ag/nylon conductive yarns as strain-sensitive elements for wearable sensor applications. A custom-built breathing simulator was employed to mimic human respiratory motion, enabling dynamic resistance measurements under cyclic strain. Conductive yarns were integrated at different structural positions to systematically evaluate their influence on mechanical durability and sensing stability. Additionally, the samples were subjected to multiple abrasion cycles to simulate cyclic wear conditions.

By analysing tensile properties, elongation behaviour, and electrical resistance stability at different abrasion levels, this study aims to identify optimal conductive yarn configurations for durable and reliable textile-based strain sensors. The findings provide valuable insights for the design and development of robust wearable textile sensors demonstrates the potential for consistent performance during the cyclic testing, providing a foundation for future more durability studies.

## Experimental work

Ten elastic band samples were produced using a COMEZ MIKRON-407^30^ crochet knitting machine, featuring a gauge of approximately 9 needles per cm, a machine width of 60 cm, and bearded-type needles, at El-Nasr Clothing and Textiles Company (KABO), Alexandria, Egypt.

**Fig. 1:**
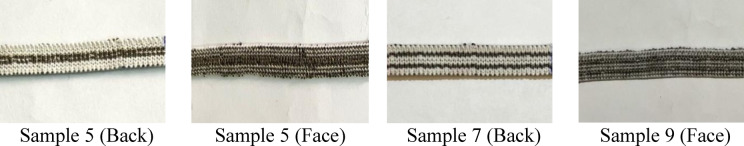
Examples of elastic band samples.

Ten elastic band samples were produced, composed of elastane yarns, polyester ground yarns, and polyester weft inlay yarns^31^. Elastane yarns made of latex rubber threads 16, which means 16 rubber threads can be inserted in one cm, providing stretch and recovery. Count of polyester ground yarns and weft inlay yarns was 150 Denier. Polyester ground yarns forming chain stitches to bind the structure and weft inlay yarns control width and enhance durability. The elastic yarns are inserted under tension and held within the crochet loops, resulting in a stable, highly extensible elastic band structure (Fig. 1). For the control sample, the elastic element consisted of 8 elastane yarns (latex rubber threads) combined with 8 polyester ground yarns. The weft structure included two face weft and two back weft polyester yarns, resulting in a balanced elastic structure. In the crochet elastic band, a conductive yarn was placed at different positions, as shown in Table 1. A Silver coated Polyamide (Nylon) fiber conductive yarn (Ag/nylon), commercial product, from WEI XING technology, Honkong, comprising 20% silver and 80% nylon. The yarn count was 3 × 70 denier, with a twist of 150 turns per meter. The conductive yarn replaced either a polyester ground yarn in the warp direction, a polyester weft yarn in the face or back, or both, allowing investigation of the effect of yarn placement on the mechanical and electrical performance of the elastic bands. Mechanical properties of the ten samples, shown in Table 1, were measured. However, the control sample was excluded from electrical property measurements due to the absence of conductive yarn.

**Table 1:** Positions of conductive yarns (C) in elastic band samples.

Sample code	Warp 1	Warp 2	Warp 3	Warp 4	Warp 5	Warp 6	Warp 7	Warp 8	Face 1	Face 2	Back 1	Back 2
Control	–	–	–	–	–	–	–	–	–	–	–	–
1	C	–	–	–	–	–	–	C	C	–	–	–
2	C	–	–	–	–	–	–	C	C	C	–	–
3	C	–	–	–	–	–	–	C	C	–	C	–
4	–	–	–	C	C	–	–	–	C	–	–	–
5	–	–	–	C	C	–	–	––	C	C	–	–
6	–	–	–	C	C	–	–	–	C	–	C	–
7	–	–	C	–	–	C	–	–	C	–	–	–
8	–	–	C	–	–	C	–	–	C	C	–	–
9	–	–	C	–	–	C	–	–	C	–	C	–

### Mechanical testing

All samples were conditioned according to the standard method (ISO 139) of textile testing for 24 h whereas the temperature and the relative humidity were (20 ± 2 and 65 ± 4%) respectively^32^. A modified Martindale (Model M235) abrasion method was employed for narrow elastic bands, treated as knitted structures. While the standard ASTM D4966^33^ is typically applied to flat fabric specimens, adaptations were necessary to suit the geometry of narrow elastic bands. Specimens were prepared according to the maximum holder diameter and fixed by stitching their ends without tension to prevent slippage while preserving their natural state. Abrasion was conducted on the back surface to simulate real use conditions, while testing of the face side is suggested for future work. Following abrasion, the pilling behaviour of the elastic band was evaluated according to ASTM D4970 (2022)^34^. Mass per unit area (gm/m^2^) was calculated by using an electronic digital balance, according to ASTM D-3776 (2017)^35^, before and after the two levels of abrasion cycles. The increase in the weight of the elastic bands was calculated after 5000 and 10,000 rubbing cycles. The percentage increase in weight was determined according to the following equation:

1 $${\text{Increase in weight \% }} = \frac{{{\mathrm{W}}2 - {\mathrm{W}}1}}{{{\mathrm{W}}1}}{*}100$$

W_1_: elastic band weight without abrasion; W_2_: elastic band weight after abrasion cycles.

The thickness of samples was measured according to ASTM D1777 (2019)^36^ by using fabric thickness tester. Moreover, mechanical and electrical properties were measured before and after applying the abrasion levels. Tensile properties, including tensile strength, elongation and tension decay, were measured using an Instron testing machine (Instron, USA), in accordance with BS EN 14704-1:2005^37^. The gauge length (75 mm), crosshead speed (300 mm/min), and specimen dimensions (200 mm in length and full width of the elastic band) were selected to ensure reliable and reproducible tensile measurements. Tension decay quantifies the reduction in tensile force over a fixed period when the band is held at a constant elongation, providing an indication to its stress relaxation behaviour. A lower tension decay % indicates higher elastic stability, reflecting the band’s ability to maintain its initial tension under prolonged stretching.

### Electronic measurement system design

**Fig. 2:**
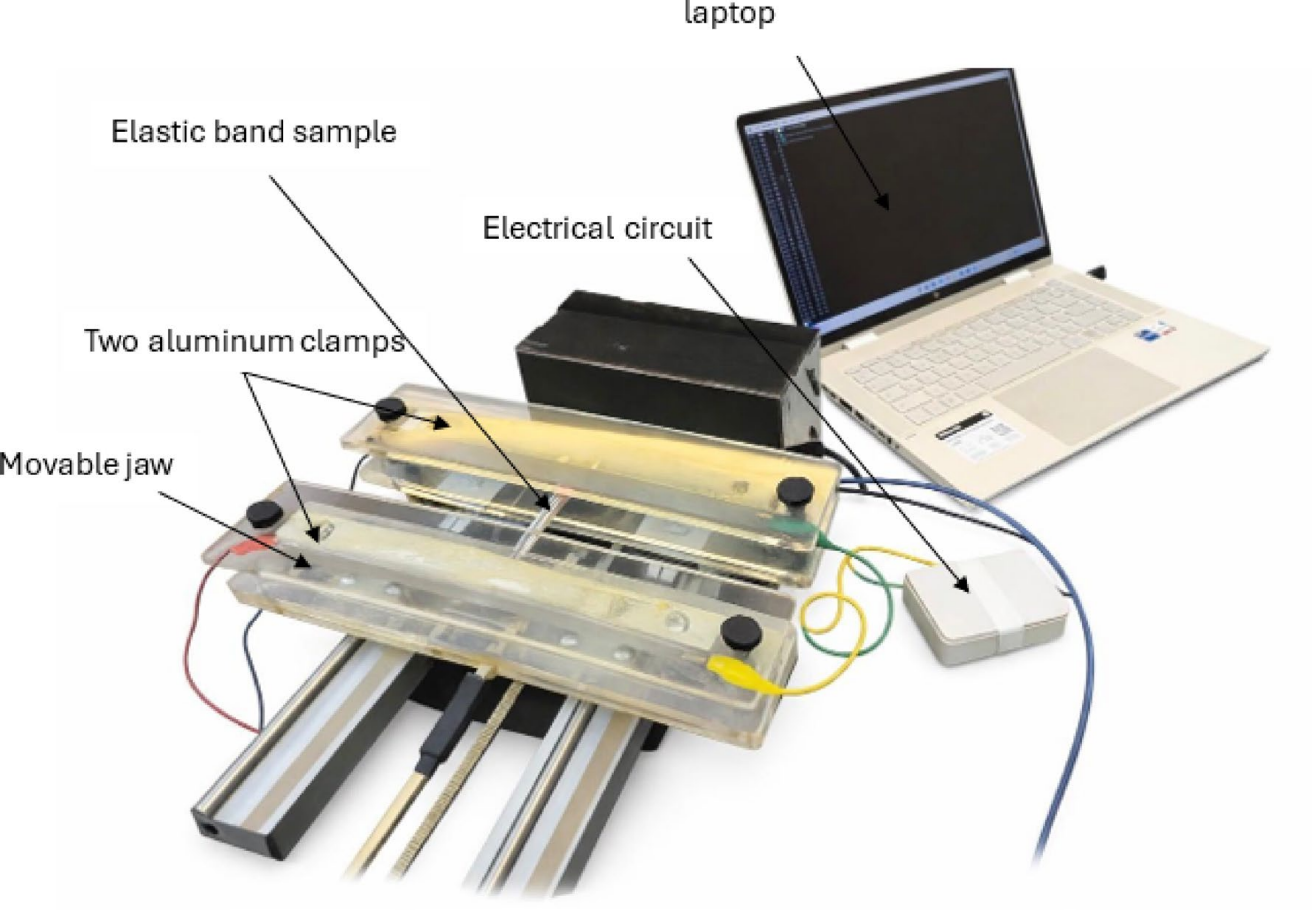
Breathing simulation system.

The measurement of electrical properties of the elastic bands based on breathing simulation system^38^ and an electrical circuit. The system comprises two aluminium clamps: one fixed and the other movable. Both clamps are used to secure the samples and enable resistance measurement. Dynamic resistance measurements were performed using a custom-built breathing simulator designed to mimic the mechanical behaviour of chest wall expansion, as shown in Fig. 2. The apparatus was calibrated following the procedures of Benha Calibration Laboratory, Egypt, in accordance with ISO 17025 requirements. It utilized a stepper motor-controlled linear actuator to apply cyclic tensile strain to the samples, which were secured between a fixed clamp and a movable jaw. The stepper motor completes one full revolution in 200 steps, and the power supply operates on AC mains of 110–240 V at 50–60 Hz. The gauge length for all samples was fixed at 10 cm. Cyclic loading was applied at a breathing rate corresponding to 5% elongation of the initial length with a time delay 1 s, following the protocol reported in^39^. Accordingly, the applied strain was maintained constant across all samples, and the resistance ratio (ΔR/R₀) was employed to compare their performance. The simulation protocol was set to a frequency of 20 cycles per minute, corresponding to the normal resting respiratory rate of healthy adults. Each measurement session lasted 30 s (approximately 10 cycles), with the ESP32 firmware acquiring data at 3.33 Hz and transmitting it wirelessly to a host computer for real-time visualization.

The ADS1115 was configured at 3.33 Hz, a rate that exceeds the Nyquist criterion for the 0.33 Hz breathing fundamental by a factor of five and is consistent with the low-power BLE transmission constraints of the wearable prototype. The primary performance indicator—the mean ΔR/R_0_ averaged across ~ 10 complete cycles per session—is robust at this sampling density; however, the authors acknowledge that higher rates would improve individual waveform fidelity.

A custom high-precision wireless measurement system was developed to acquire resistance data during dynamic deformation. The architecture is anchored by an ESP32 microcontroller (Espressif Systems), selected for its dual-core processing and integrated Bluetooth Low Energy (BLE) capabilities essential for real-time wearable data transmission. To address the resolution limitations of standard microcontrollers, a 16-bit analog-to-digital converter (ADS1115, Texas Instruments) was integrated via I2C protocol. Operating with a programmable gain amplifier and a configurable sampling rate, the ADS1115 provides significantly superior resolution compared to built-in 12-bit ADCs.

**Fig. 3:**
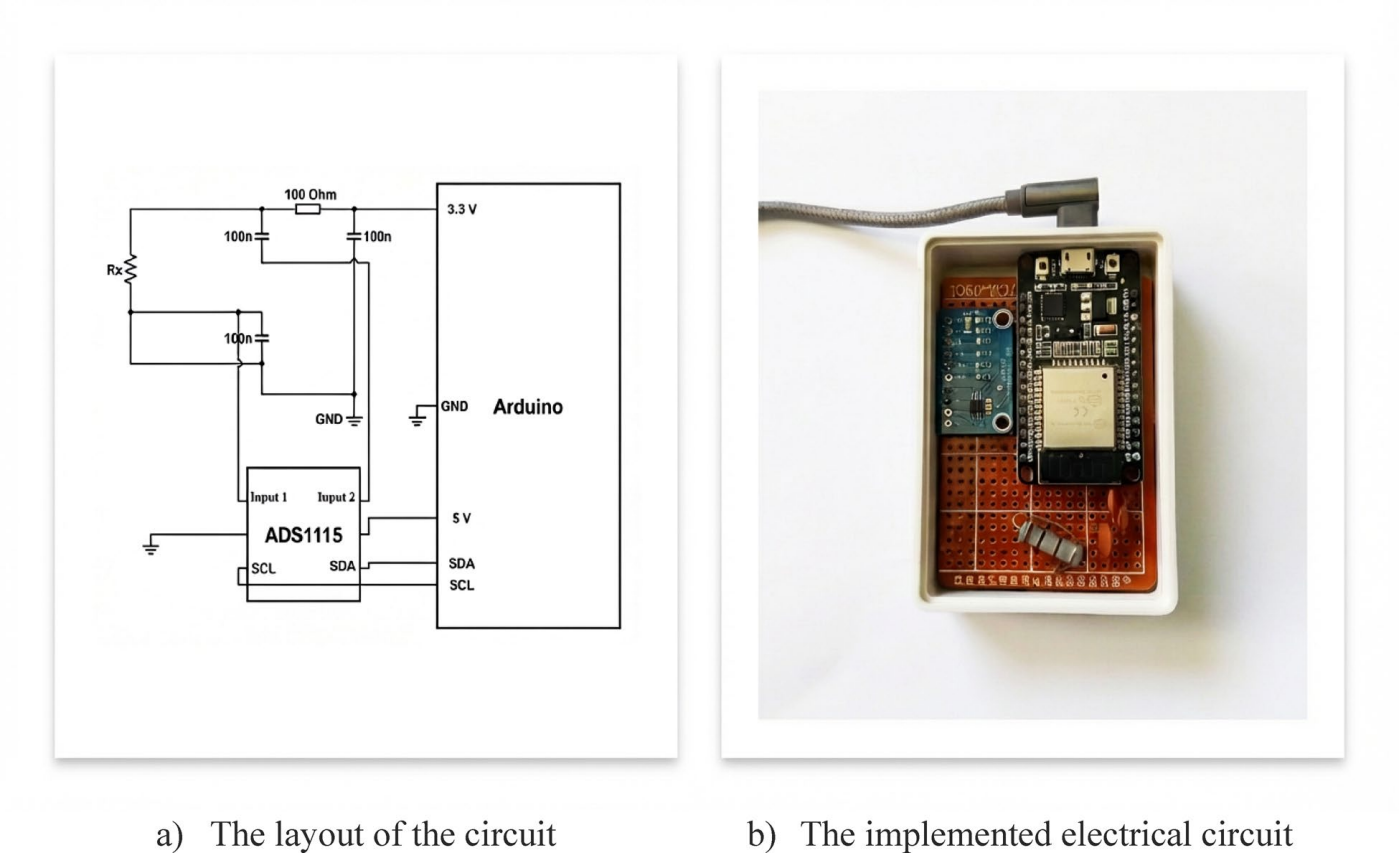
The electrical circuit and its layout.

The circuit employs a voltage divider configuration powered by a regulated 3.3 V supply, where the elastic sample is connected in series with a precision 100 Ω (± 1%) reference resistor, as shown in Fig. 3. The ADS1115 measures the voltage drop in differential mode, enabling accurate resistance calculation via Ohm’s law while minimizing errors from supply fluctuations. To ensure signal integrity, hardware filtering was implemented using 100 nF ceramic capacitors at strategic analog input and power nodes. These low-pass RC filters attenuate high-frequency noise while preserving the respiratory signal bandwidth (0.2–0.5 Hz). The system is powered by a 10,000 mAh portable power bank to provide consistent regulation and eliminate ground loop interference.

The electrical contacts were established through direct clamping using wide-surface aluminium clamps, which were selected to maximise the conductor-contact area and reduce contact resistance at the sample interface. This configuration constitutes a two-wire (2W) measurement setup, appropriate for portable wearable prototypes where simplicity and low power consumption are primary design constraints. The relative resistance metric ΔR/R₀ employed throughout this study substantially mitigates the influence of any stable contact resistance component, as this term cancels when computing the difference R(t) − R_0_. The baseline R₀ was re-established at the commencement of each measurement session to account for session-to-session variability in contact conditions.

### Data analysis and statistical methods

#### Statistical performance analysis

Data obtained from different testing methods were analysed both statistically and graphically. Bar charts were used to illustrate the effect of various variables on the mechanical, aesthetic and electrical properties. A two-way ANOVA with replications was performed to evaluate the simultaneous effects of conductive yarn position and different abrasion cycles on the measured properties. Five samples were tested for each property to ensure statistical reliability, and the results were statistically significant (*p* < 0.05) at a 95% confidence level. Moreover, Python 3.13 was employed to process and analyse the electrical property data, including the generation and interpretation of electrical graphs to evaluate the samples’ performance.

#### Signal preprocessing and performance metrics

Raw voltage measurements acquired from the ADS1115 at 3.33 Hz during the 30-s breathing simulation were converted to resistance values using Ohm’s law and the voltage divider equation.

The baseline resistance R_0_ for each sample was defined as the initial resistance measured at the relaxed state before the start of the breathing simulation. The instantaneous relative resistance change at any given time point **t** was computed as:

2 $$\Delta {\mathrm{R}}\left( {\mathrm{t}} \right)/{\mathrm{R}}_{0} \left( \% \right) = \left[ {\left( {{\mathrm{R}}\left( {\mathrm{t}} \right) - {\mathrm{R}}_{0} } \right)/{\mathrm{R}}_{0} } \right] \times {1}00$$

where R(t) represents the measured resistance at time t.

For each sample after applying abrasion conditions by Martindale (Without abrasion, 5000 cycles, 10,000 cycles), the sensor response was quantified by calculating the arithmetic mean of the relative resistance change values over the selected steady-state period. Performance retention was quantified to assess durability as:

3 $${\mathrm{Retention}}\;\left( \% \right) = \left[ {\left( {{\mathrm{Mean}}\;{\mathrm{Response}}} \right)\;{\mathrm{at}}\;{1}0,000\;{\mathrm{cycles}}/\left( {{\mathrm{Mean}}\;{\mathrm{Response}}} \right)\;{\mathrm{at}}\;{\mathrm{without}}\;{\mathrm{abrasion}}} \right] \times {1}00$$

Values exceeding 100% indicate performance enhancement after abrasion, whereas values below 100% denote performance degradation.

## Results and discussion

### Mechanical properties

**Fig. 4:**
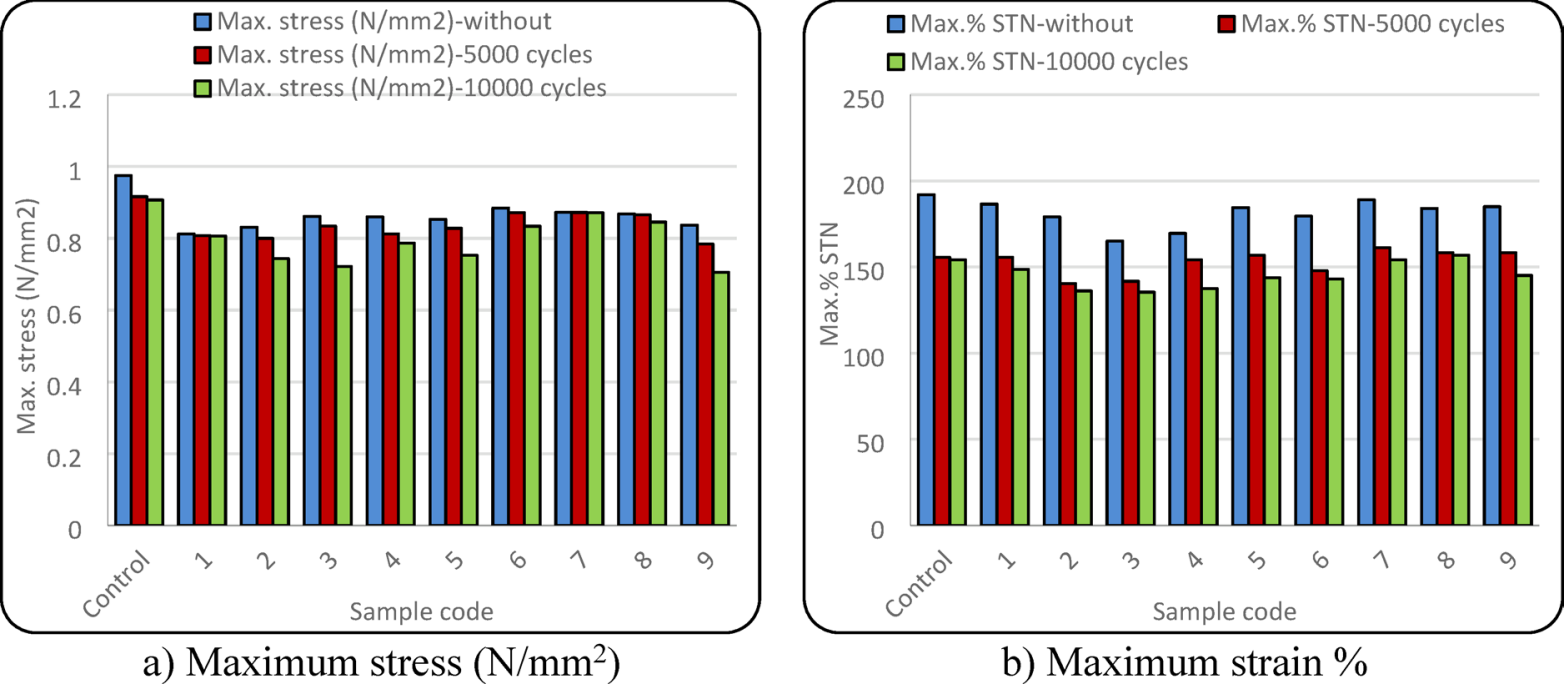
Mechanical properties of elastic band samples.

Table 2 illustrates ANOVA results of the effect of the position of conductive yarns and abrasion cycles on mechanical properties of elastic bands. The position of the conductive yarn has a statistically significant effect on both tensile stress and strain%. The p-values are less than (α = 0.05), indicating that the observed differences among the samples are not due to random variation. Figure 4 illustrates the tensile performance of the elastic bands in terms of the values of maximum stress and maximum strain%. The samples were strongly influenced by both the position of the conductive yarn and the applied abrasion cycles. The control sample, which contained no conductive yarn, exhibited the highest tensile stress and elongation values under all testing conditions. In contrast, the degree of reduction observed in the other samples relative to the control varied depending on the position of the conductive yarn within the elastic structure and the applied abrasion cycles. This behaviour is attributed to the absence of conductive yarn, which maintains a homogeneous elastic structure, allowing the applied tensile load to be evenly distributed without mechanical discontinuities^40–42^. Regarding the effect of abrasion cycles, increasing the cycles led to a reduction in tensile stress and elongation% due to cumulative surface damage and partial loss of elastic recovery^43,44^.

**Table 2:** P-values of ANOVA analysis for fabric properties.

Characteristic	ANOVA *p*-values	
	C-yarn position	Abrasion cycles
Max. stress (N/mm^2^)	0.000	0.000
Max strain %	0.000	0.000
Tension decay %	0.172	0.0003
Increase in weight %	0.029	0.0007
Pilling grades	0.13	0.0003

**Fig. 5:**
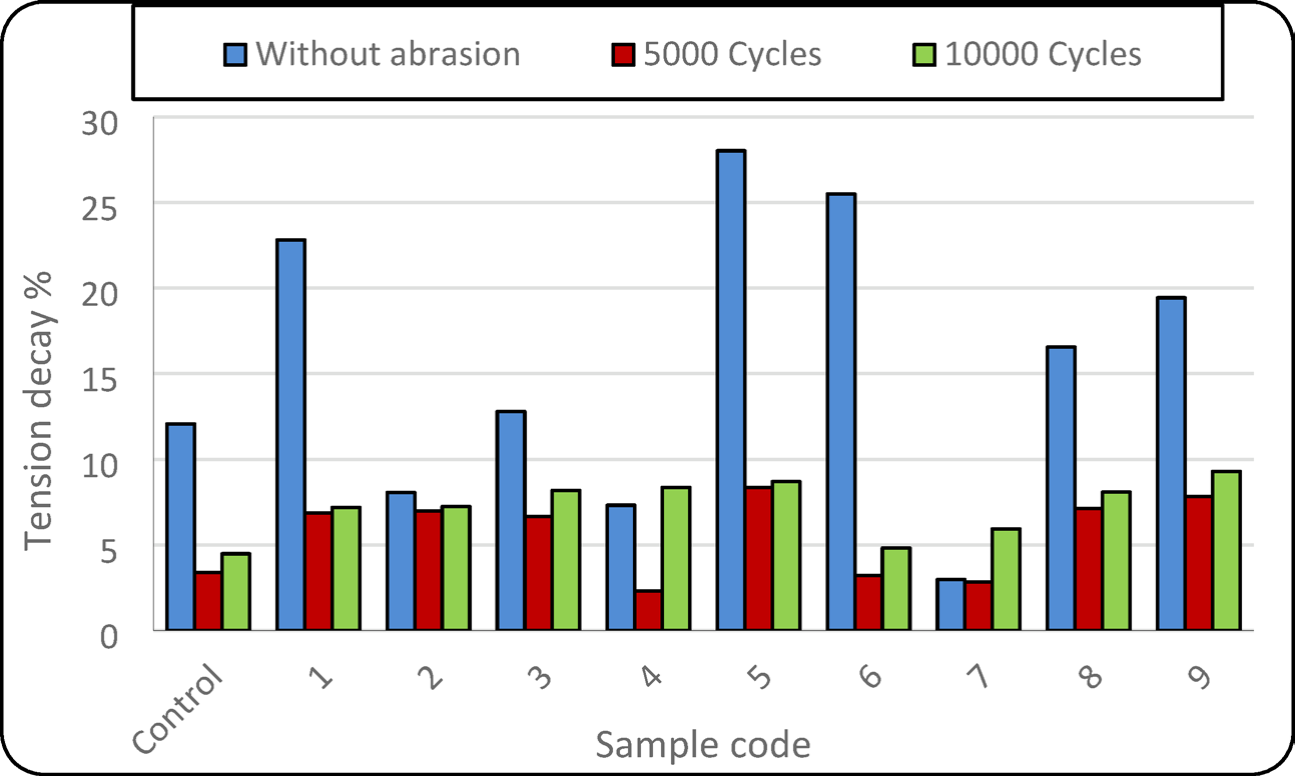
Tension decay% at the different levels of abrasion cycles.

Table 2 demonstrates that the number of abrasion cycles has a statistically significant effect on the tension decay behaviour of the elastic bands, as indicated by a p-value of 0.0003, which is well below the significance level (α = 0.05). This confirms that repeated abrasion substantially influences the loss of elastic recovery. In contrast, the position of the conductive yarn does not exhibit a statistically significant effect on tension decay. The reduction in tension decay, as shown in Fig. 5, observed after 5000 abrasion cycles is associated with increased structural compactness, which limits yarn mobility and reduces effective elongation. With further abrasion up to 10,000 cycles, accumulated mechanical damage slightly increases tension decay due to partial degradation of elastic components. Differences among samples are mainly related to the position of conductive yarns, which affects stress distribution and internal friction within the elastic band. However, abrasion cycles play a more dominant role in controlling tension decay behaviour than conductive yarn positioning.

### Aesthetic properties (Increase in weight % and pilling grades)

After 5000 and 10,000 abrasion cycles, aesthetic properties were evaluated, represented by pilling grades and appearance properties.

After applying abrasion cycles, the weight of elastic bands increased due to pilling formation. Table 2 presents the results of the two-way ANOVA analysis conducted to evaluate the effects of conductive yarn position within the elastic band and the number of abrasion cycles on both weight increase (%) and pilling grades. For weight increase (%), the conductive yarn position exhibited a statistically significant effect, as indicated by a p-value of 0.029 (*p* < 0.05). In contrast, its effect on pilling grades was not statistically significant (*p* = 0.13). Abrasion cycles showed a highly significant influence on both responses, very low p-values (0.0007 and 0.0003, respectively).

**Fig. 6:**
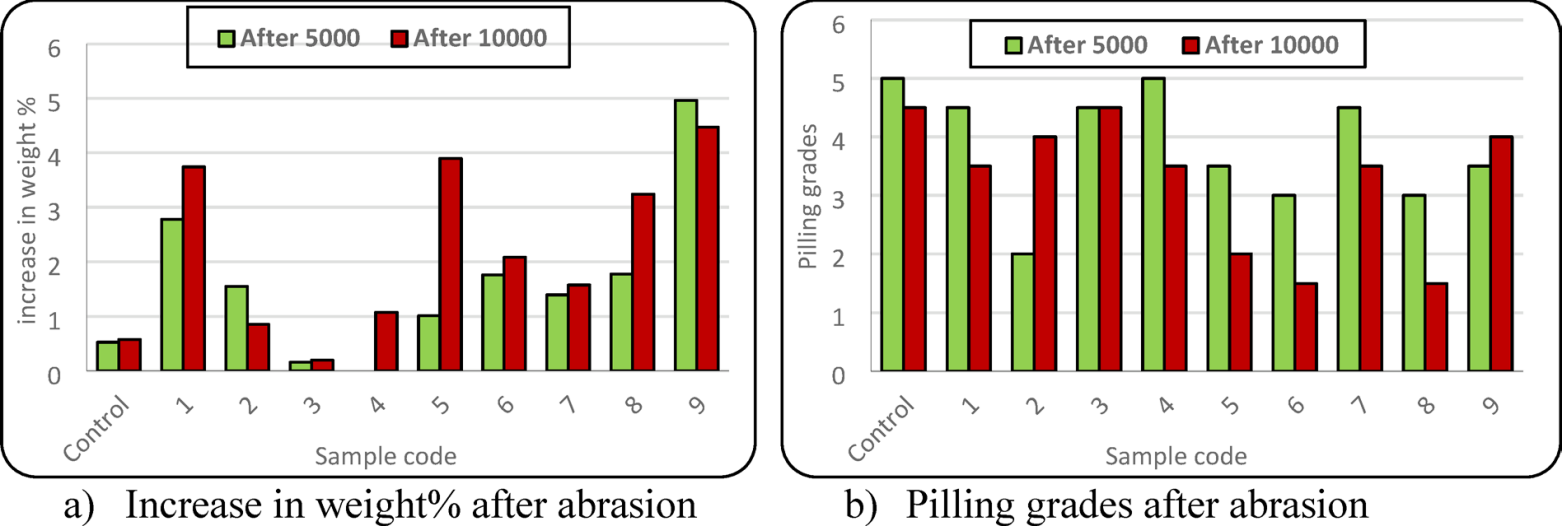
Effect of fabric variables on weight and pilling grades.

Figure 6 illustrate the effect of conductive yarn position within the elastic band and the number of abrasion cycles on weight increase (%) and pilling grades. For all samples, abrasion resulted in a noticeable increase in weight after both 5000 and 10,000 cycles except for sample (4) at 5000 cycles. The higher increase in weight indicates the higher pills formation, whereas the lower weight increase corresponds to reduced pilling. The increase in weight is mainly attributed to fiber migration and accumulation on the elastic band surface during abrasion and adhering to the fabric surface, forming pills which are promoted by the abrading medium during abrasion testing. In some samples, the increase of weight decreased after 10,000 abrasion cycles, which was accompanied by an improvement in pilling grades. This behaviour is attributed to the break-off and removal of previously formed pills due to abrasion process^45^.

**Fig. 7:**
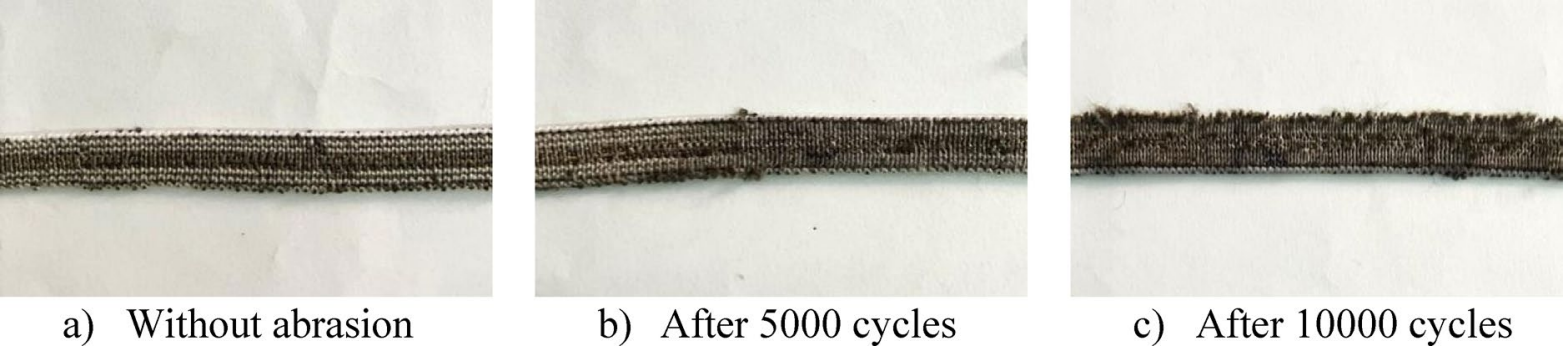
Deterioration of elastic band surface due to abrasion (Sample 5).

Table 3 and Fig. 7 illustrate the effect of abrasion cycles and conductive yarn positioning on the surface condition and thickness of the elastic bands. As abrasion progressed from the unabraded state to 5000 and 10,000 cycles, a gradual deterioration of the fabric surface is observed, characterized by increased surface roughness and the formation of pills, as particularly illustrated for Sample 5. In some samples, thickness increased after abrasion due to fiber protrusion, pill formation, and material accumulation on the surface, while in other cases a reduction was observed, which can be attributed to fiber breakage, surface flattening, and material loss under repeated rubbing. While the control sample initially exhibited the smallest overall variation, according to the difference between thickness in different conditions, analysis of the experimental samples containing conductive yarn revealed that sample 3 displayed the least changes in both abrasion conditions. This indicates that sample 3, which incorporates conductive yarn, demonstrated the highest stability among the tested functional samples. Overall, the combined visual and thickness data confirm that abrasion alters the surface morphology of the elastic bands, resulting in fluctuating thickness behaviour.

**Table 3:** Thickness of elastic band samples.

Sample code	Without	5000 cycles	10,000 cycles
Control	1.284 ± 0.005	1.29 ± 0.014	1.294 ± 0.01
1	1.257 ± 0.012	1.241 ± 0.016	1.285 ± 0.021
2	1.278 ± 0.046	1.315 ± 0.004	1.258 ± 0.018
3	1.238 ± 0.018	1.247 ± 0.003	1.264 ± 0.013
4	1.178 ± 0.046	1.25 ± 0.028	1.26 ± 0.007
5	1.257 ± 0.006	1.238 ± 0.018	1.355 ± 0.021
6	1.275 ± 0.1	1.216 ± 0.02	1.23 ± 0.025
7	1.255 ± 0.018	1.215 ± 0.007	1.286 ± 0.016
8	1.262 ± 0.009	1.253 ± 0.011	1.325 ± 0.021
9	1.294 ± 0.005	1.276 ± 0.022	1.326 ± 0.028

### Electrical properties

#### Dynamic signal integrity and electrical stability

**Fig. 8:**
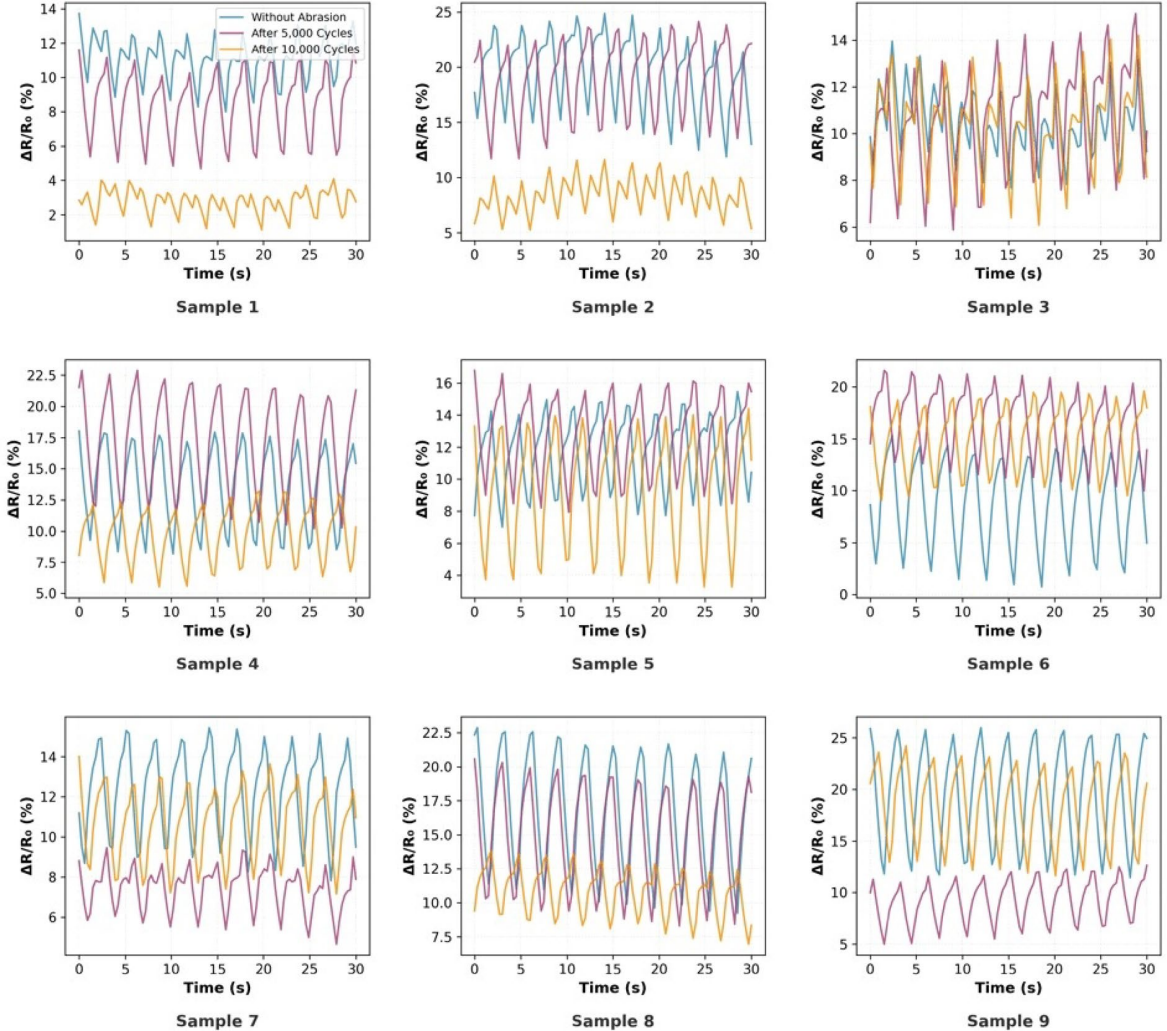
Temporal ΔR/R_0_ patterns under simulated breathing and abrasion.

Figure 8 presents representative time-series data illustrating the dynamic resistance changes (ΔR/R_0_) during simulated breathing cycles for all nine samples across the three test conditions. The temporal patterns reveal consistent periodic behaviour corresponding to the 20 cycles per minute breathing rate, with a clear distinction between inhalation (increasing resistance) and exhalation (decreasing resistance) phases. Samples without abrasion treatment generally exhibited well-defined sinusoidal patterns with minimal baseline drift.

The impact of abrasion on signal quality varied systematically across samples. Sample 1, after 10,000 cycles, exhibited significantly dampened oscillation amplitude (mean ΔR/R_0_ = 2.73% vs. 11.11% without abrasion), yet it maintained relatively stable periodic behaviour. Conversely, Sample 6 demonstrated increased oscillation amplitude (8.66% without abrasion to 16.80% at 5000 cycles) while maintaining signal stability. Sample 9 maintained high-amplitude, stable oscillations across all abrasion conditions. Among all samples, sample 3 demonstrated the highest stability in its electrical properties, showing minimal variation in signal characteristics under the three different treatment conditions.

#### Baseline sensing characteristics and statistical performance

The quantitative analysis of the resistance change performance revealed variation across the nine sample configurations. Table 4 presents the experimentally measured resistance values (R) of all samples under different abrasion conditions.

**Table 4:** Resistance values (R) of samples under different abrasion conditions.

Sample	Without abrasion	After 5000 cycles	After 10,000 cycles
S1	23.48 ± 0.25	20.55 ± 0.35	28.01 ± 0.19
S2	15.36 ± 0.4	16.18 ± 0.44	17.35 ± 0.23
S3	15.66 ± 0.19	13.64 ± 0.26	14 ± 0.23
S4	15.75 ± 0.29	15.61 ± 0.49	16 ± 0.3
S5	15.1 ± 0.56	17.11 ± 0.35	16.99 ± 0.5
S6	15.32 ± 0.28	17.13 ± 0.44	17.33 ± 0.44
S7	15.57 ± 0.53	16.34 ± 0.16	18.09 ± 0.28
S8	12.79 ± 0.5	17.77 ± 0.55	17.73 ± 0.26
S9	23.48 ± 0.25	15.14 ± 0.27	13.23 ± 0.39

**Fig. 9:**
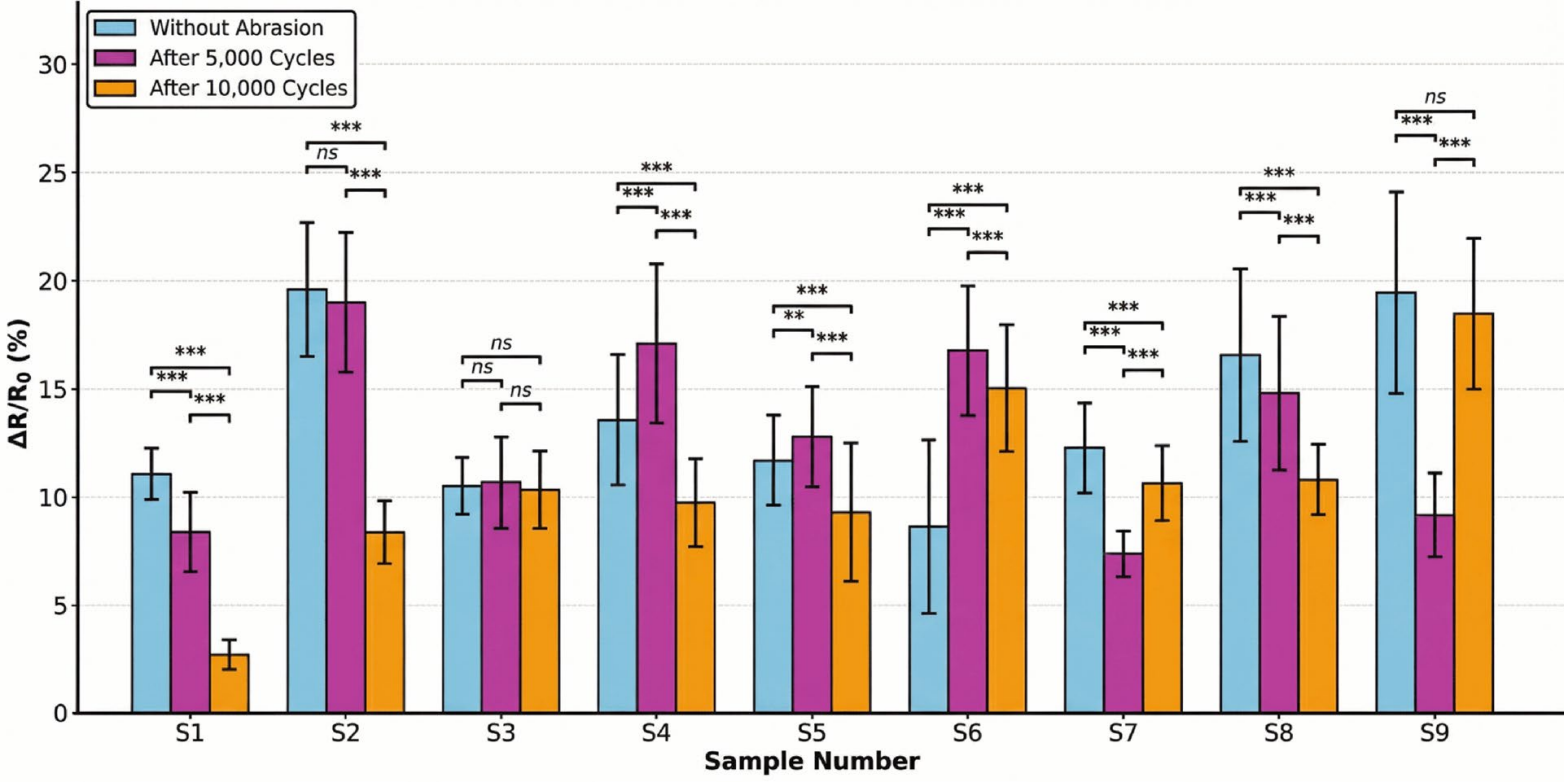
Statistical comparison of (ΔR/R₀) for conductive bands. (*p-value < 0.05, **p-value < 0.01, ***p-value < 0.001 and ns: nonsignificant, *p* > 0.05).

As detailed in Table 5 and Fig. 9, the mean relative resistance changes of without abrasion samples ranged from 8.66 to 19.62%. Sample 2 demonstrated the highest sensitivity (19.62%), while sample 6 exhibited the lowest (8.66%).

**Table 5:** Performance summary of conductive elastic band samples (ΔR/R_0_%).

Sample	Without abrasion	After 5000 cycles	After 10,000 cycles
S1	11.11 ± 0.82	8.42 ± 0.65	2.73 ± 0.21
S2	19.62 ± 1.45	19.04 ± 1.38	8.40 ± 0.62
S3	10.54 ± 0.78	10.70 ± 0.79	10.38 ± 0.76
S4	13.59 ± 1.01	17.13 ± 1.25	9.77 ± 0.72
S5	11.73 ± 0.87	12.83 ± 0.94	9.32 ± 0.69
S6	8.66 ± 0.64	16.80 ± 1.23	15.06 ± 1.11
S7	12.30 ± 0.91	7.40 ± 0.54	10.66 ± 0.79
S8	16.59 ± 1.22	14.82 ± 1.09	10.83 ± 0.80
S9	19.46 ± 1.44	9.18 ± 0.68	18.49 ± 1.36

The strain gauge factor (GF) represents the sensitivity of the conductive elastic band to mechanical deformation, expressed as the ratio of relative resistance change (ΔR/R_0_) to the applied strain (ε). Based on the data in Table 6, a clear variation in gauge factor values can be observed before and after abrasion cycles (5000 and 10,000 cycles), reflecting the influence of mechanical wear on electrical performance. From a practical and comparative standpoint, the most desirable behaviour is stability of the strain gauge factor across different abrasion levels, as this indicates consistent sensing performance and structural integrity of the conductive network within the sample.

**Table 6:** Strain gauge of conductive elastic band samples (ΔR/R_0_/ε).

Sample	Without abrasion	After 5000 cycles	After 10,000 cycles
S1	222.2	168.4	54.6
S2	392.4	380.8	168
S3	210.8	214	207.6
S4	271.8	342.6	195.4
S5	234.6	256.6	186.4
S6	173.2	336	301.2
S7	246	148	213.2
S8	331.8	296.4	216.6
S9	389.2	183.6	369.8

Among the tested samples, S3 shows the most stable behaviour, with values (210.8, 214, and 207.6) remaining nearly constant despite increasing abrasion. Such stability reflects the material’s ability to maintain consistent electrical response under repeated mechanical stress, which is a key requirement for practical strain-sensing applications. In contrast, samples such as S1 and S2 exhibit significant reductions in gauge factor after abrasion, indicating deterioration in their sensing capability.

**Fig. 10:**
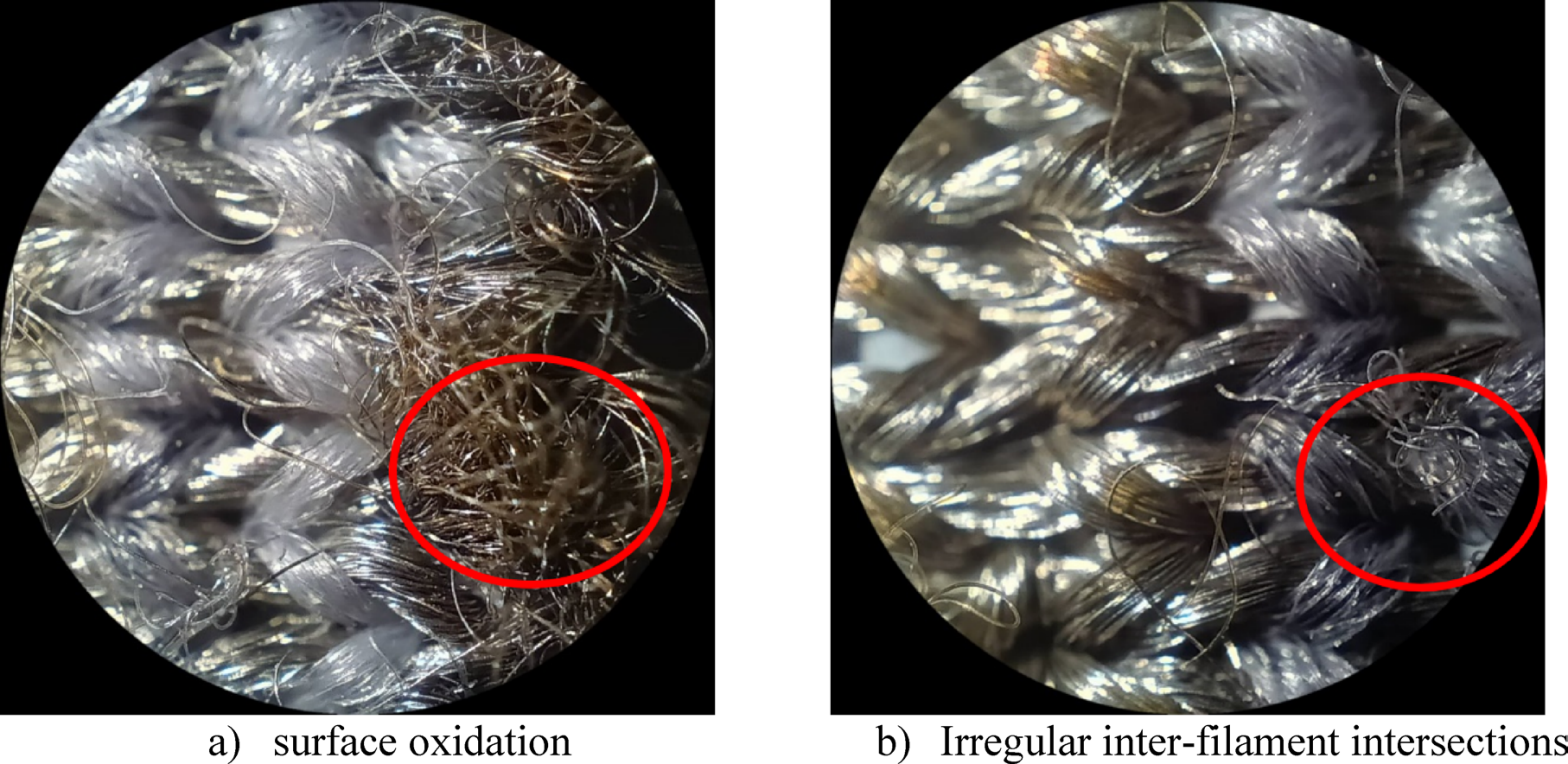
Evolution of the conductive elastic band structure after Abrasion.

Furthermore, the baseline resistance (R_0_) evolution provided insights into the internal structural changes, as shown in Table 7. Sample 1 experienced a significant 29.0% increase in R₀ (from 21.13 to 27.27 Ω) after 10,000 cycles, suggesting that the increase may be attributed to surface oxidation of the conductive yarns, which reduces the number of parallel conductive paths, consistent with the discoloration (yellowish to reddish-brown) observed in Fig. 10. Conversely, samples maintaining stable baseline resistance with non-significant effect (*P* > 0.05), such as sample 3 (− 10.4% change), demonstrated superior structural integrity with well-protected conductive elements. This observation is further supported by Figs. 8 and 9, which indicate a relatively stable behaviour under abrasion conditions.

**Table 7:** Baseline resistance (R_0_) evolution under abrasion.

Sample	Without abrasion (Ω)	After 5000 (Ω)	After 10,000 (Ω)	Resistance change (%)
S1	21.13	18.95	27.27	+ 29.0
S2	12.84	13.59	16.01	+ 24.7
S3	14.16	12.32	12.69	− 10.4
S4	11.88	13.33	14.58	+ 22.7
S5	14.09	15.17	15.54	+ 10.3
S6	13.90	14.67	15.06	+ 8.3
S7	13.64	15.21	16.35	+ 19.9
S8	13.36	15.48	16.00	+ 19.8
S9	10.71	13.87	11.16	+ 4.2

#### Abrasion-induced performance evolution

**Fig. 11:**
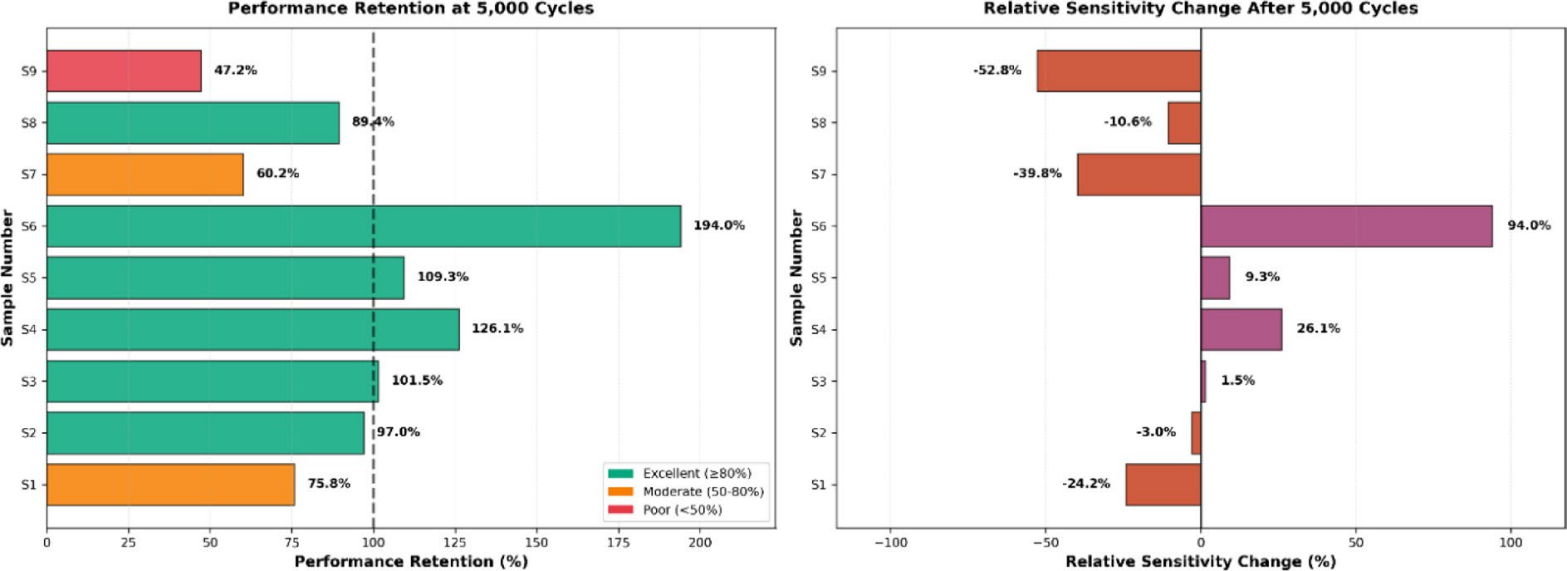
Performance retention and relative sensitivity change after 5000 cycles.

**Fig. 12:**
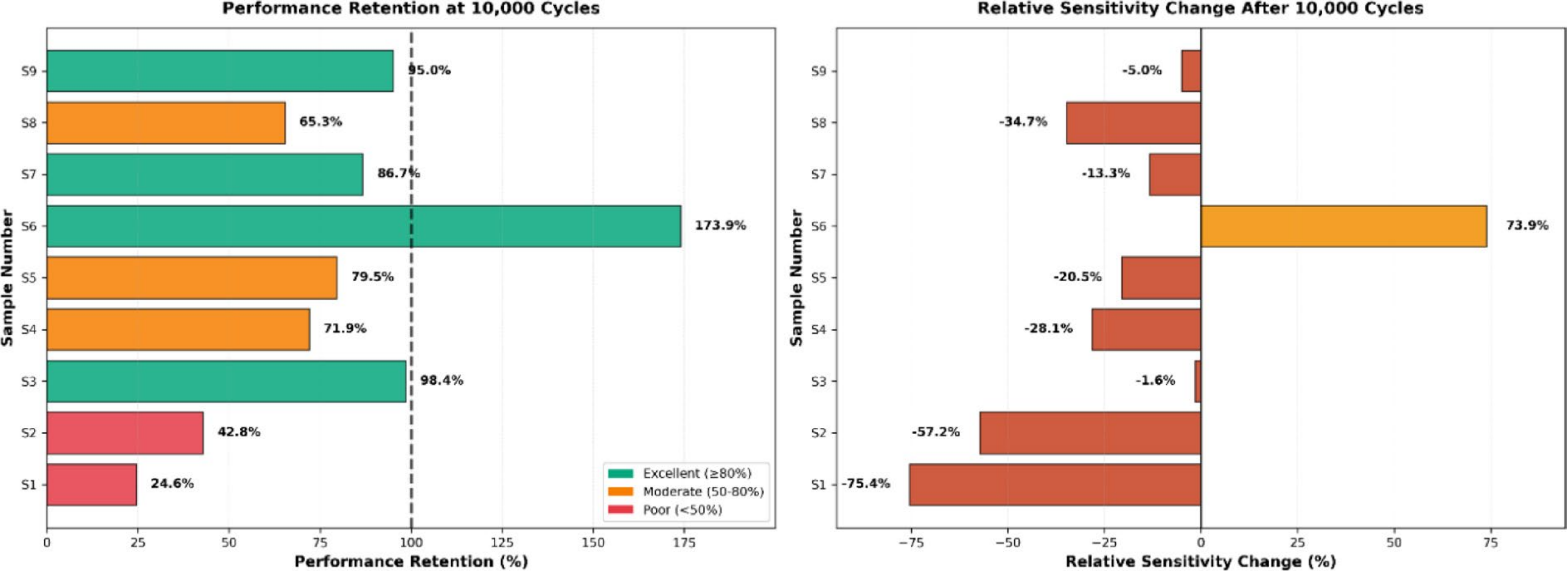
Performance retention and relative sensitivity change after 10,000 cycles.

The temporal evolution of sensor performance under abrasion stress revealed distinct physical degradation mechanisms, which can be categorized into three response patterns based on the 5000 cycles and 10,000 cycles, as shown in Figs. 11 and 12.

- Vulnerable and delayed-failure patterns: samples with predominantly warp-based conductive paths exhibited severe deterioration but with different temporal dynamics. Sample 1 showed a continuous, linear degradation pattern, with performance decreasing to 75.8% after 5000 abrasion cycles and further declining to 24.6% after 10,000 cycles. In contrast, sample 2 displayed a “delayed failure” mechanism; it maintained excellent stability through the first 5000 cycles (97.0% retention) before experiencing a catastrophic breakdown by 10,000 cycles (42.8% retention), suggesting that its structure resists initial wear but fails rapidly once critical conductive paths are severed.
- Robust and stable patterns: sample 3 demonstrated exceptional durability, emerging as the most robust design. It exhibited negligible performance loss, maintaining 101.5% retention at 5000 cycles and stabilizing at 98.4% after 10,000 cycles, confirming that its balanced warp-weft integration effectively protects the conductive network.
- Anomalous enhancement and self-recovery: several samples exhibited non-monotonic behaviours driven by surface modifications. Sample 6 showed a paradoxical “performance enhancement,” where sensitivity nearly doubled (194.0% retention) at 5000 cycles and remained elevated at 10,000 cycles (173.9%), likely due to abrasion-induced irregular inter-filament intersections and disoriented surface morphology after abrasion, as shown in Figs. 7 and 10. Most notably, sample 9 and sample 7 displayed a “self-recovery” pattern. sample 9 dropped significantly to 47.2% retention at 5000 cycles but remarkably recovered to 95.0% by 10,000 cycles. similarly, sample 7 improved from 60.2% (5000 cycles) to 86.7% (10,000 cycles), suggesting a structural reorganization of conductive fibers that re-establishes electrical pathways during extended wear.

## Conclusion

This study presents a comprehensive evaluation of conductive elastic bands as wearable textile strain sensors, with a particular focus on the combined effects of conductive yarn configuration and abrasion-induced mechanical wear. By employing a dynamic testing approach that mimics human respiratory motion, the electrical properties of conductive elastic bands were measured after applying different abrasion conditions. The experimental results demonstrate that abrasion significantly influences the mechanical integrity, surface morphology, and electrical performance of the elastic bands. While abrasion generally led to reduced tensile properties and surface deterioration, the extent of electrical degradation varied markedly depending on the structural placement of the conductive yarns. It is worth noting that, sample (3) exhibited outstanding durability, maintaining stable baseline resistance and consistent sensing response even after 10,000 abrasion cycles.

The observed differences in performance are attributed to variations in stress distribution, inter-yarn friction, and the protection of conductive pathways within the elastic structure. These findings confirm that conductive yarn positioning is a decisive design parameter governing the cyclic reliability of elastic band sensors.

Overall, this work provides important insights into the design of durable and reliable wearable strain sensors and demonstrates that elastic band structures, when optimally configured, can sustain stable electrical performance under simulated physiological motion and cyclic mechanical abrasion. The outcomes of this study offer a practical foundation for advancing wearable textile sensors for continuous respiration monitoring and related healthcare applications.

## Limitations

This study is limited to short-term cyclic testing under controlled breathing simulation. No washing durability testing was performed. These aspects are recommended for future work. Furthermore, the resistance measurement system employed a two-wire voltage-divider configuration; for absolute resistance values in the 12–28 Ω range, a four-wire (Kelvin) sensing arrangement would eliminate the contact resistance contribution entirely and is recommended for future instrumentation-focused studies. Additionally, the ADS1115 acquisition rate of 3.33 Hz yields approximately 10 data points per breathing cycle, which is sufficient for computing the mean ΔR/R₀ reported herein but limits single-cycle waveform reconstruction fidelity; higher sampling rates (≥ 10 Hz) are recommended in future work requiring detailed waveform morphology analysis.

## Data Availability

The datasets used and/or analysed during the current study available from the corresponding author on reasonable request.
